# Comparative analysis of the social vulnerability assessment to climate change applied to fisheries from Spain and Turkey

**DOI:** 10.1038/s41598-021-93165-0

**Published:** 2021-07-06

**Authors:** Mauro Gómez Murciano, Yajie Liu, Vahdet Ünal, José Luis Sánchez LIzaso

**Affiliations:** 1 Department of Marine Science and Applied Biology, University of Alicante, Alicante, Spain; 2 Faculty of Bioscience, Fisheries and Economics, UiT The Arctic University of Norway, Tromsø, Norway; 3 Faculty of Fisheries, Ege University, Izmir, Turkey

**Keywords:** Climate-change adaptation, Climate-change impacts, Climate-change policy, Socioeconomic scenarios

## Abstract

The aim of this study is to assess the climate effects on fisheries from a bottom-up approach based on fishers’ fishing experience, knowledge, and perceptions. To perform this task, a social vulnerability assessment was conducted in two different fishing areas: one in Spain and the other one in Turkey. The vulnerability was measured using the collected data and information through a structured questionnaire, and surveys were carried out among fishers in the Castelló (Spain) and the Aegean Sea (Turkey) between 2018 and 2019. Overall, the results indicated that the two studied regions have a moderate to high vulnerability and that the Aegean Sea was slightly more vulnerable than Castelló. It was also found that storms and temperature are the main climatic stressors that affect the fishing sector, and the economic indicators such as revenue from fishing in both regions showed high degrees of sensitivity. To reduce the vulnerability to climate change, adaptive measures should be implemented while taking into consideration the specific socio-economic and institutional characteristics of each region. In conclusion, the effects of climate change on the fishing sector and their social vulnerability are diverse. Consequently, there is no single climate measure that can minimize the vulnerability of fishing sectors in different regions.

## Introduction

Coastal and marine ecosystems provide a variety of goods and services, including provisioning, supporting, regulating, and cultural services^1^. Humans directly and indirectly depend on these services for their livelihoods and wellbeing. However, many marine ecosystems are increasingly under anthropologic and environmental pressure due to overfishing, pollution, habitat destruction, and environmental degradation^2,3^. Climate change is generally accepted as one of the major issues that face human societies in the twenty-first century. Global climate change affects the atmospheric and oceanic systems of the Earth, and it has widespread and varying effects on many marine ecosystems and their services due to the rise of sea temperature and sea level, melting of sea ice, loss of oxygen, and acidification^3,4^. Obviously, the warming of oceans has led to changes in the marine productivity, community composition, and ecosystem structure, consequently shifting the abundance, distribution, and composition of fish species through growth, reproduction, and survival^4–6^.

In the last decades, climate-driven changes have intensified, especially in Polar Regions, which poses a serious threat to marine species and ecosystems. The warming temperatures have pushed tropical species to higher latitudes, so marine species have declined in warm-water regions and increased in cold-water polar regions^6^. These changes affect the availability of resources to fisheries, which puts fishing communities at a high risk of climate change. Many economies and people depend on fishing resources when it comes to sustaining their nutritional needs, livelihood, and wellbeing^7^. However, global marine fisheries are now economically underperforming due to unsustainable fishing, pollution, and habitat degradation. Added to these threats is the looming challenge of climate change^8^.

Climate change challenges the management of contemporary fisheries in many parts of the world and gives rise to significant additional ecological and socio-economic uncertainties^9^. In the fishing sector, integrating the climate effects into the management of fisheries has become increasingly important for decision making and planning. Also, the effects of climate change on fishing practices and the adaptive capacities of fisheries have become significant factors for implementing management measures and for taking actions to cope with the effects of climate change^10^. Nevertheless, such effects and adaptive capacities vary based on a range of factors, including the fishing location, targeted species, received income, and institutional support. It is acknowledged that climate change is a gradual and long-term climate process^11^. The adaptive responses to climate change have evolved, and now some societies respond to environmental change while maintaining their livelihood and wellbeing by utilizing natural resources, such as fisheries. However, different individual resource users and resource-dependent communities have different abilities and capacities to cope with and adapt to climate change. Such differences may be due to an assortment of factors, including access to information and resources, the skills and knowledge of different communities, social and human capital, social connections, and social-demographics^12,13^.

The Mediterranean Sea is one of the world’s most valuable seas, and it has been a hotspot of climate change. The warming water, decreasing precipitation, increasing heatwaves, and extreme weather events have caused many species to shift their distribution westwards and northwards^14^ and led to the establishment of new habitats for some species, such as meridionalization (occurrence of warm water species in northern regions) and tropicalization (expansion of non-native tropical species)^15^. The rising temperatures aid the establishment of foreign species in the Mediterranean, which are mostly introduced via the Suez Canal, and climate change likely facilitates their success in the Mediterranean^16–18^. The fish resources in the Mediterranean Sea have been rapidly declining due to a combination of overfishing, habitat destruction, marine pollution, invasive species, and climate change^19–21^. Some invasive species cause great economic losses to fishers, as they compete with native species for food and habitat. For instance, the economic loss of fishers in the Eastern Mediterranean, which is mainly caused by the silver-cheeked toadfish (*Lagocephalus sceleratus*), has been increasing over the years^22–24^.

Also, the Mediterranean fisheries are highly heterogeneous in terms of harvested species, employed fishing fleets, complex socio-economic elements, and governance capacities in riparian countries^15^. The dominant fishing fleets in the Mediterranean Sea are small-scale boats defined by vessel size, and most are polyvalent vessels (i.e., using more than one type of gear) that can have a length of up to 12 meters. Such fisheries comprise up to 80% of the total number of fishing fleets and employ most of the fishers^25^. In contrast, the other 20% of fishing fleets comprise trawlers and purse seiners, of which trawlers harvest species with the highest landed values while purse seiners have the highest landings but with lower landed values^26^. Therefore, in many respects, small-scale fisheries in the Mediterranean region are quite important and cannot be ignored, making them highly vulnerable to the negative impacts of climate change^15,27^. Climate change has complex and diverse impacts on marine resources^28^. On one hand, climate change poses a serious challenge to fisheries and fishers for their livelihood. On the other hand, it also presents a potential opportunity for developing new fisheries resulting from the emerging resources that did not previously exist^29–31^.

The overarching goal of this study is to assist decision-makers at both the use of resources and policymaking to identify and verbalize the components that would enable the fishing sector to develop appropriate adaptation measures that can address the effects of climate change on fishing communities and fishers. Thus, the capacity of the fishing sector can be enhanced at the local level so that they will take part in the development of climate adaptation plan and action. The main objective of this study is to assess the social vulnerability of the fishing industry to the impacts of climate change based on two case studies in the Mediterranean Sea. Two contrasting case studies were selected to represent different fishing fleets and fishing practice in the Eastern Mediterranean with small-scale fisheries and in the Western Mediterranean with diverse fleet characteristics. A bottom-up participatory approach from the fishers’ perspective was employed, and data were collected through a structured questionnaire and face-to-face interview surveys with fishers were conducted in two selected fishing areas. This study may contribute to the overall scope of improving the resilience of fisheries and fishers, reducing their vulnerability to climate change, and enabling fishing communities and decision makers to respond in a timely manner to the changes in the marine ecosystems caused by climate changes^13^.

## Results

A total of 27 indicators were selected to assess the social vulnerability in the two selected regions (Table 1). Two climate stressors (temperature and storms) were included as exposure, 9 indicators as sensitivity and 16 indicators as adaptive capacity. 46 fishers from Castelló in the Eastern Mediterranean Sea, and 85 fishers from the Aegean Sea in the Western Mediterranean Sea were interviewed and responded our questionnaire. It should be noted that the results are interpreted based on the defined scales and scores in this paper which are between 0.00 and 1.00 as low, 1.01 and 2.00 as moderate, 2.01 and 3.00 as high and 3.01 and 4.00 as very high (see Table 2).

**Table 1:** Vulnerability levels and selected indicators.

Levels	Indicators	Answer Choices (scale 4–1) 4—very high, 3—high, 2—moderate, 1—low
**Exposure (2)**		
Climate factors	Temperature	4
	Storms	3
**Sensitivity (9)**		
Economic	Revenues from fishing	100%—4; 75%— 3; 50%—2 < 25%—1
	Revenues from others	< 25%—4; 50%—3; 75%—2 100%—1
	Private consumption of catch	< 5%—1; 10%—2; 15%—3 > 20%—4
	Change in Catch composition	Yes, lost dominant species—4 Yes, new dominant species—3 Yes, new mix of species—2 None—1
	Change in catch	Decreased—4; Stable—3 Increased—2; N/A—1
Fisheries	Change in fishing gear	Yes—3; No—1
	Causes of change in fishing gear	Change in target species—4 Change in ocean conditions—3 Changes in labour/law—2 Others or No, like safety—1
	Change in harvest species	Yes—3; No—1
	Change in fish size	Yes, become smaller—4 No change—3 Yes, become bigger—2 N/A—1
**Adaptive capacity (16)**		
Social demographic	Alternative opportunities	No—4; Agriculture—3 Relocation/jobs in the urban areas—2 Aquaculture—1
	Financial support	No—4; Others (occasional)—3 Private—2; Community—1
	Governmental support for losses from fishing	100%—1; > 50%—2 < 30%—3; Little or none—4
	Family size	> 5—4; 4—3; 3 –-2; 2 or 1—1
	Professional training	School—1; Family—2 Friends and self-learn—3; No—4
	Income	Average scores of revenue from fishing and others
	Financial support for other trainings if fishing stops	Government—1 Union or association—2 Self—3; None—4
	Education level	College—1; High school—2 Secondary school—3 No formal schooling—4
	Fishing years	> 20 years—4; 10 -20 years—3 5–10 years—2; < 5 years—1
Institutional	Government capacity	Very high level—1; Good—2 Somehow good—3; Little or none—4
	Fishers direct involvement with policy	Always—1; Mostly—2 Sometimes ---3; Never—4
	Regulation enforcement	Always—1; Mostly—2 Only sometimes—3 Little or not at all—4
	Fairly applied rules	100%—1; Mostly—2 Sometimes—3; Not at all—4
	Government responses to changes	100%—1; Mostly—2 Sometimes—3; Not at all—4
	Transparency and trust	100%—1; Mostly—2 Sometimes—3; Not at all—4
	Insurance and health care	100%—1; Mostly—2 Sometimes—3; Not at all—4

**Table 2:** The levels defined based on the ranges of the index scores for climate stressors, sensitivity, adaptive capacity and cumulative vulnerability^66,72^.

Levels	Score range
Very high	3.01–4.00
High	2.01–3.00
Moderate	1.01–2.00
Low	0.00–1.00

### Exposure

In all cases, the most relevant climatic factors that were identified as threats by fishers were storms and temperature that have relatively high effects on both regions (Table 3). They were also identified from different studied areas as the greatest threats to fishing activities. The fishers of the Aegean Sea and the artisanal fleet of Castelló indicated that storms are the climatic factor that mostly affects their fishing activities, and that temperature comes second. However, in the regions from the north to the south, the temperature has a stronger effect on purse-seine and trawling fleets than storms.

**Table 3:** Vulnerability from the fishing areas: Castelló and Aegean.

	Exposure	Sensitivity	Adaptive capacity	Cumulative vulnerability
Castelló	3.05	1.94	2.72	2.27
Aegean	3.28	2.27	2.57	2.98

The scores for exposure, sensitivity and adaptive capacity dimensions are calculated using Eq. (1)^58,67^, and cumulative vulnerability score is calculated with Eq. (2)^58^.

### Sensitivity

Both regions have moderate to high sensitivity to climate change according to established scoring although the Castelló fishing region (1.94) shows slightly lower sensitivity than the Aegean fishing regions (2.27) (Table 3). In the Castelló fishing region, the most sensitive indicators include “Revenues from fishing”, “Change in fish size”, “Causes of change in fishing gear” and “Changes in harvest species”. In the Aegean Sea area, the most sensitive indicators consist of “Revenues from fishing and others” and “Catch composition”. Of which, the “Revenues from fishing” is perceived as the most sensitive indictor to the fishing sector in both regions. In the Aegean Sea the “Revenues from others” indicator is as important as the “Revenues from fishing” indicator. They have a direct negative–positive relationship (Table 4). In other words, when the importance of one of them increases, the importance of the other one decreases. It is not the case for the Castelló fishing region. This is because over 95% of the Castelló fishers directly generate their income from fishing, while only 16% of the fishers in the south and central Aegean regions get 100% of their income from fishing, and 22% of the fishers’ income is from other activities. Only a few fishers of the Aegean Sea have a direct income from fishing, and most of the fishers in the Aegean Sea complement their incomes with other jobs, as they also work, such as taxi drivers, street vendors, maintenance managers. The difference in the received fishing income in these two regions is also reflected by the “Consumption of catch”. The fishers of the Aegean region are the ones who consume most of the fish from their catches, as they can consume about over 20% of their catches annually in comparison with the Castelló fishers, who only consume less than 5%.

**Table 4:** Component matrix for the sensitivity indicators from PCA loading.

Indicators	PC 1	
	Castelló	Aegean
**Economic dimension**		
Revenues from fishing	0.75	− 0.90
Revenues from others	0.07	0.88
Private consumption of catch	− 0.24	0.43
Catch composition	0.13	0.49
Change in catch	− 0.14	− 0.13
**Fisheries**		
Change in fishing gear	0.29	− 0.27
Causes of change in fishing gear	− 0.64	− 0.05
Change in harvest species	0.59	0.30
Change in fish size	0.69	0.03

In parallel, the “Change in fishing gear” and “Causes of change in fishing gear” indicators also have a direct negative–positive relationship In the Castelló region (Table 4). Fishers in this area consider these indicators as the highly susceptible to the threats of climate change. Fishers do not see the need to modify their fishing gear if the gear they use is still allowed and effective. In all the regions, most fishers continued to fish with the same fishing gear they have always used to carry out their daily fishing activities. In the case of Castelló, those who did so were, for the most part, trawlers. In the case of the Aegean region, around 40% of the fishers changed their gear during the last 10 years, and 60% kept using the same fishing gear. The 42% of the fishers who changed their gear were from the northern region. The reasons why fishers have decided to change their fishing gear in recent years are diverse. Although most of the fishers began to use different gear due to changes in their regional laws and regulations, some changed their gear due to changes in the conditions at sea, practical reasons (maintenance problems, worn-out gear, old gear, damaged gear, hard-to-use gear), and safety.

In terms of the total catches, there were big differences in the two regions. 18% of the fishers in the Aegean Sea and 52% of those in the Castelló region revealed that their catches have remained unchanged in the last decade. However, 69% and 35% of the fishers in the Aegean and Castelló, respectively, reported that their catches had decreased during the last 10 years. With respect to the catch composition, 46% and 44% of the fishers in the Aegean Sea and the Castelló region, respectively, indicated that their catch composition had not changed in the last decade. 9% and 30% of the fishers in the Aegean Sea and the Castelló region, respectively, reported that their catches comprised of mixed species, while 26% and 13% of the fishers in the Aegean Sea and the Castelló region, respectively, stated that their catches were dominated by other species other than the species they used to catch.

With the presence of new species, such as pufferfish (e.g., *Lagocephalus sceleratus*) and rabbitfish (*Siganus luridus*), fishing in the Aegean Sea has been seriously harmed, as these species directly affect many fishing activities and therefore the fishers’ income. In comparison, the Castelló fleets do not appreciate the modification in the species they catch. What causes the lower flow of income in the Castelló area is the decrease in the amount of caught fish.

### Adaptive capacity

According to our definition, the fishing sectors in both regions have shown a high adaptive capacity for climate change. However, the Castelló fishers have marginally higher adaptive capacity (2.72) than the Aegean fishers (2.57) (Table 3). The “Governmental response to change” and “Transparency and trust” were claimed as the most factors affecting the social adaptive capacity, especially for the Castelló fishers. On one hand, without counting their earnings from their profession, fishers in the Castelló do not receive any other kind of financial support. On the other hand, fishers in the Aegean Sea do receive some financial assistance from the government and others. For inshore fleets in both regions, governments do not provide any help if the fishers’ catches are reduced, and when there was available aid, it only covers less than half of their fishing expenses. Except for the fishers of purse-seine fleets, the other fishers stated that they did receive a percentage of income from their governments as compensation for the reduction in the amount of caught fish.

Each region uses different measures based on the needs of their fishers. In the case of the Castelló region, the “Professional training”, “Insurance” and “Education” are the indicators that guarantee fishers to keep carrying out their fishing activities. They can indicate the fishers’ adaptive abilities for the future changes in the fishing sector in these regions. In both cases, the level of formal education of fishers is relatively low. Many fishers have just primary school, while some have never been schooled. This data is more striking in artisanal fleets. In the Aegean region, 7% of the fishers have college education and 27% have high school education. The fishers also implied that there is no training available if they wanted to change to other professions. If they are interested in learning new professions, they must pay all the costs themselves.

At the same time, in the Aegean Sea, the “Fairly applied rules”, “Government capacity” and “Regulation enforcement” indicators enable the administrations of fisheries to deal with new threats faster and can enhance the adaptive capacity for different fishers (Table 5). Most of the fishers (71%) from the Aegean Sea fleets stated that they feel they can share information with their fishing authorities. However, 47% of the fishers in Castelló stated that they can share information with their authorities and 35% of them stated that they cannot. In all the regions, over half of the fishers consider that their authorities do not have the capacity to determine appropriate regulations for fisheries. Further, less than half of the fishers believe that there is no enforcement of the laws and regulations. However, the fishers in the southern and northern Aegean regions believe that rules and regulations are usually or sometimes enforced. In contrast with the central region, they think that laws are inadequately enforced or that there is no enforcement at all. 59% of the fishers in Castelló perceived that rules and regulations are usually enforced, especially for artisanal and trawling fleets. Additionally, experienced fishers in the Aegean Sea show higher adaptive capacity than less experienced fishers.

**Table 5:** Component matrix for the adaptive capacity indicators from PCA loading.

Indicators	PC 1	
	Castelló	Aegean
**Social-demographic dimension**		
Alternative opportunities	0.44	− 0.05
Financial support	− 0.07	0.29
Governmental support for losses from fishing	− 0.08	− 0.30
Family size	− 0.36	− 0.42
Income	0.40	0.46
Financial support for other trainings if fishing stops	0.37	− 0.32
Fishers' direct involvement in policy	− 0.05	0.30
Professional training	0.67	− 0.03
Education level	0.60	0.07
Fishing years	0.41	0.64
**Institutional dimension **		
Government capacity	0.32	0.71
Regulation enforcement	0.03	0.71
Fairly applied rules	− 0.22	0.78
Government responses to changes	0.93	0.68
Transparency and trust	0.93	0.66
Insurance and health care	0.79	0.49

### Vulnerability

The Aegean region in the eastern Mediterranean Sea (2.98) has higher cumulative vulnerability than the Castelló region (2.27) in the western Mediterranean Sea. In both regions, the institutional, social-economic and climatic factors have the highest variability. Regarding the social-demographic factors, the responses were more unanimous in the fishing fleets in each region.

**Figure 1:**
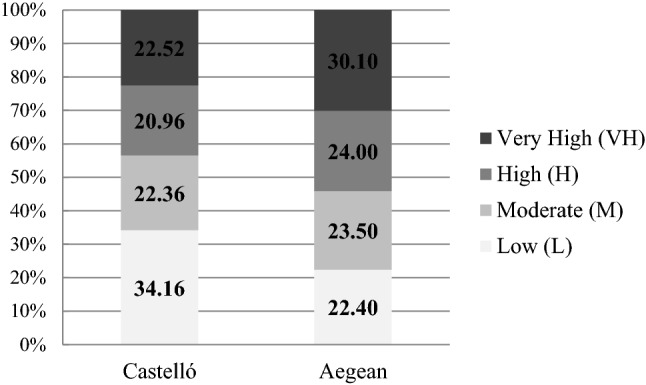
Distribution of the respondents by the vulnerability levels.

For the individual respondents, the vulnerability assessment score showed a relatively even distribution of the respondents under various levels of the vulnerability assessment in two different regions (see Eqs. 1, 2 in material and methodology section). While 30% of the respondents scored a very high vulnerability level in the Aegean region, 34% scored a low vulnerability level in the Castelló region. Most of the respondents scored 22% on average under the other levels (Fig. 1).

Overall, the fishing sector in these two regions has a moderate to high vulnerability, and the fishers in the Aegean region (2.98) were found to be more vulnerable than in the Castelló region (2.27). The fishers in the Aegean region are more exposed and sensitive than those in the Castelló region. In contrast, the Castelló region has higher adaptive capacity than in the Aegean region. The score for all the groups was different for each indicator, indicating different exposure, sensitivity, and adaptive capacity levels. The average of these indicators was employed to calculate the vulnerability level in two studied areas.

## Discussion

The Castelló (Spain) and Aegean Sea (Turkey) regions showed a moderate to high level of cumulative vulnerability. The Castelló region has a relatively lower vulnerability level than the Aegean region. Since there are different approaches used for assessing social vulnerability with respect to climate stressors^7^, the resulting quantitative measures such as scores may differ. It should be noted that our results are interpreted based on the scales defined and indicators used here. However, regardless of scoring methods and indicators used, the assessed cumulative vulnerability in this study is in line with those previously assessed by other studies^7,9,15,16,32^. In other words, fisheries in the study areas show moderate to high vulnerability to climate change. This study assessed the social vulnerability of fisheries to climate change from a bottom-up approach based on the fishers’ fishing experiences, knowledge, and perceptions. While the top-down approaches were closely associated with the climate change impact assessment and emerged in a large part from the risk/hazard analysis on the vulnerability, the bottom-up approaches were closely associated with the political economy/ecology tradition and the livelihoods perspective on vulnerability^7^. The bottom-up approaches are directly associated with participatory stakeholders (social-system) while the top-down approaches are relied on biophysical system (ecological system) and indirectly related to stakeholders^7^. The exposure, sensitivity, and adaptive capacity influence the vulnerability of fishery-based livelihoods in varied ways. Those that are most exposed are not necessarily the most sensitive or least able to adapt^28^. Studies that specifically assess the synergistic effects of both climate change and fishing on the fishery resources and ecosystem functioning in the Mediterranean Sea are rare^33^, and very limited studies have projected the consequences of climate change on marine ecosystems in an integrated way in the Mediterranean^14,34^.

In this study, a number of variables (indicators) were used and indexed for analyzing the vulnerability. These variables were developed based on some previous studies (details see “Material and methods”). For instance, Ebert et al.^35^ and Durlyapong and Nakhapakorn^36^ developed a socio-economic vulnerability index for climate change, and it is composed of four variables. Ahsan and Warner^37^ used the same index and increased the number of variables to analyze the affected communities in south-western coastal Bangladesh. Further, face-to-face interviews with fishers were employed for collecting data and information. These participatory stakeholder-based interactive interviews typically exemplify bottom-up/qualitative methodologies^7^. This approach is more time and labor-intensive, but there is a very small chance to have missing data from surveys^37^, so it has proven to be very suitable for such studies. Then, the data was analyzed using Principal Component Analysis, which was indicated by the study of Tadić et al.^38^ as an especially useful tool for conducting such analysis. Particularly, a study that has many variables (e.g., the Mediterranean region) shows high sensitivity to climate.

In the two case study areas, it was found that storms and temperature are the main climate stressors affecting the fishing sector, and this situation may be intensified in the near future. Generally, a rapid increase is expected in the marine temperature, sea level, and frequency of storms, which would increase the negative impact on the fishing activities in the Mediterranean Sea^39^. The effects of climate change may also increase the intensity and size of weather events^7^. At a global scale, one of the main effects of climate change on marine ecosystems is the change in the rate and patterns of primary production^40^, which directly affects the fishing productivity. Eventually, on average, the richness in species and the catch potential of fisheries are projected to an increase at mid and high latitudes and to a decrease at tropical latitudes^11^. Finally, in response to climate change and intensive fishing, widespread reductions in the sizes of fish and in the mean size of zooplankton have been observed over time, and these trends further affect the sustainability of fisheries^41^.

Another effect of climate change is that of invasive species. In the Aegean Sea, *Lagocephalus sceleratus* was first observed by Mouneimne^42^, and *L. sceleratus* was first observed in 2003 off Akayka, Gökova Bay in Turkey^43^. In recent years, the invasive marine fish *L. sceleratus* (silver-cheeked toadfish) had the biggest impact on both the local species and the socio-economic well-being of fishers^22,23,44^. Other species like *Saurida undosquamis* are also invading the Mediterranean. The presence of these new species combined with overfishing has changed the catch composition of many fisheries, as mentioned by the fishers who took the survey. However, some of these invasive fish species may present economic values^39^. To fight this situation, the Akyaka fishing cooperative (Gökova bay) started selling Brushtooth lizardfish to control its population and increase its economic value. However, the market acceptance of new species is likely to culturally vary with the location^45^. In the Aegean Sea, local people do not know about *S. undosquamis* and they are not comfortable with buying it. Thus, it is unclear whether these species provide an opportunity as a climate-adaptive measure to the fishers of the Mediterranean.

The susceptibility of fishing communities to the effects of climate change depends on the importance of fishing in relation to other occupations^46^. Fishing communities might respond to changes in marine systems using a variety of ways, including outmigration, where young people move to other communities^47^, and changes in economic activities, markets, and/or trading patterns^48^. For instance, most of the small-scaled fishing activities in the Aegean Sea have negative or insufficient economic performance^19,49^, and most of the fishers complement their income with other jobs to support their livelihood. Contrarily, the present study indicates that more than half of the fishers earn almost all their income from fisheries, especially in the Castelló region. However, if fishers cannot go fishing anymore or decide to stop their fishing activities, their options are limited. This study indicated that 25% of the Aegean fishers expressed their desire to quit their professions. However, they remain in business as it provides self-employment, and they continue fishing due to the lack of any alternative opportunities^19^. Nevertheless, in many Mediterranean countries, a person wishing to be a professional fisher must have the necessary skills^49^. For example, in Spain, a fisher must have a navigation/fishing certificate showing that he has the necessary skills for working in the fishing sector^50^. Most of these fishers are people with limited training and are specialized in the maritime-fishing sector. Thus, it would be exceedingly difficult for them to find new jobs outside the fishing sector. It should be noted that the fishing fleet is rather old with an average age of over 45 years. The lack of skilled fishers and the aging vessels are expected to become the main threats that will make fishing more vulnerable in the short and medium term. There are a few young people who are willing to take over their parents and grandparents as fishers, but this is not something unusual. This trend also brings with it a decline in artisanal fisheries in many coastal zones, and this situation is leading to losses in the traditional and ecological knowledge of fishers^51^.

Finally, the Castelló and Aegean Sea regions present a moderate to high vulnerability level. In contrast, the relative vulnerabilities of the economic sectors of Europe, North America, and Australia to the impacts of climate change regarding their fisheries are low in comparison with other regions^32^. In Europe, an adaptation policy was developed at the international, national, and local governmental levels, including the prioritization of adaptation options^41^. In simple terms, local level actions can help reduce the vulnerability of coastal communities to the impacts of climate change. There are no mechanisms for including climate data in the assessments of fisheries or fishery-relevant data in climate models, although the institutional potential for doing so exists^45^. Therefore, it is vital to know how climate change directly affects fishers, how vulnerable they are and what adaptive capacity they have. Using approaches like the one in this study (bottom-up methodology), appropriate adaptive strategies can be prioritized and developed to meet the actual needs of fishers. Also, the implementation of new regulations is urgently needed to preserve the livelihood of professional fishers (with a well-defined professional identity) whose livelihoods entirely depend on fishing^19^. In addition, the management strategies and measures, which reduce vulnerability and promote resilience, can change the status quo for many agencies and institutions, but they are frequently resisted^52^. At present, the policies and measures in both studied regions do not consider the necessary ecosystem approaches or tools for mitigating climate change.

## Conclusion

The cumulative vulnerability level was moderate-high in the case of Castelló and high in the case of the Aegean Sea. The sets of indicators used for assessing social vulnerability in the studies areas have different implications for designing climate management and policies. The present study indicated that storms and temperature as “Climate factors” are the most important indicator, which is perceived as the main threat by fishers in all the studied regions. Therefore, fishing authorities should consider management strategies to mitigate or adapt climate impacts that are caused by them. Since the “Fishing revenues” indicator showed the highest sensitivity in the studied regions, specific indicators for each region should be considered in the design and implementation of policies. Also, the conducted vulnerability assessment helped structure how we think about the ways through which climate change affects fishers, and the used framework helped identify and organize the opportunities and challenges of dealing with such problems. This study is just a beginning, which means that the adaptation to climate change and other global environmental change is an iterative process that still requires both top-down and bottom-up processes.

## Material and methods

### Vulnerability assessment framework

**Figure 2:**
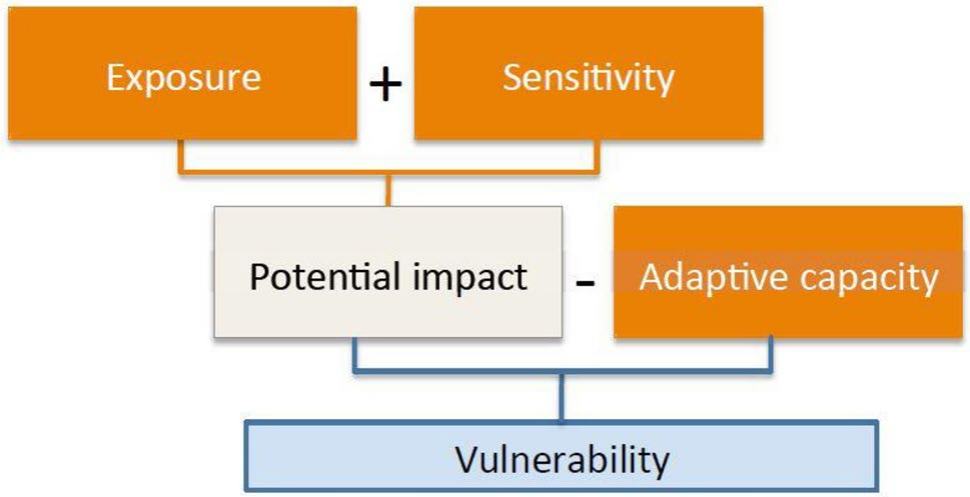
Conceptual model of the vulnerability components ( Source: FAO, 2015).

According to the IPCC, the vulnerability is defined as the propensity or predisposition to be adversely affected^28^. Another definition can be “*The degree to which a system is susceptible to, or unable to cope with, adverse effects of climate change, including climate variability and extremes. Vulnerability is a function of the character, magnitude, and rate of climate variation to which a system is exposed, its Sensitivity, and its Adaptive Capacity*”^53^. The vulnerability, in the context of social and environmental changes, is defined as the state of susceptibility to be harmed from perturbations^52^, especially from climatic shocks^37^, and it consists of three well-defined components or dimensions^7^ (Fig. 2): (1) exposure, (2) sensitivity, and (3) adaptive capacity. The exposure is defined as “*The degree to which a system is stressed by climate stimuli, such as the magnitude, frequency, and duration of a climatic event such as temperature anomalies or extreme weather events*”^53^, and it can also be interpreted as a social-ecological system and its associated ecosystem services that may be adversely affected by climate stressors, such as temperature, acidification, storms, etc.^28^. The sensitivity and adaptive capacity are defined as “*The ability of* a social-ecological *system to adjust to climate change (including climate variability and extremes) to moderate potential damages, to take advantage of opportunities or cope with the consequences*”^53^, and they can also be seen as the intrinsic degree to which the biophysical, social, and economic conditions may be influenced by extrinsic stresses or hazards^28^. These three components are interrelated and independent, so their relevance and interpretation depend on the scale of analysis, the particular sector under consideration, and data availability^54,55^. The exposure and sensitivity are closely related and are determined by environmental and social forces, while the adaptive capacity is shaped by different cultural, social, economic, and institutional forces^56^. The sensitivity refers to *the susceptibility* of a social system (e.g., people or communities), which is either negatively or positively affected by climate stressors^52^. The sensitivity of social systems depends on the economic, political, cultural, and institutional factors that allow buffering or the attenuation of change^46^.

Global climate change has effects on various social–ecological systems and their associated ecosystem services. The vulnerability and adaptive capacity greatly vary across social–ecological systems at spatial and temporal scales as well as across related ecosystem services at global and local levels. Some researchers have conceptualized, interpreted, and applied vulnerability measures in different ways and for different policy purposes^32,52,56–58^. Vulnerability assessments can be conducted through top-down or bottom-up approaches^59^. Using model-generated climate data, the top-down approach is widely used on the ecological part of the social–ecological systems focusing on biophysical vulnerability. However, at a particular location and for a particular sector or industry, it is more appropriate to use the bottom-up approach that focuses on the social part of social-ecological systems at the household, industry and community levels.

The social vulnerability is to examine and understand the human use of resources, and it mainly focuses on the ability of resource users to respond (i.e., to cope and adapt) to climate change^60^. This study aims at assessing the social vulnerability of the fishing sector while focusing on local fishers through using a participatory approach that is solely based on the fishers’ opinions, knowledge, perceptions, and practical experiences. Thus, this paper analyzes the social vulnerability from a socio-economic perspective based on the fishers’ views and on their ability to adapt to climate change.

The vulnerability is a relative measure, and there are no absolute measures that can be observed and measured. Thus, this study identifies and selects a number of indicators (Table 1) representing the Exposure, Sensitivity, and Adaptive Capacity to climate change. These indicators were selected and analyzed based on biological, economic, social-demographic, and institutional perspectives. First, we examined which climate stressors fishers are exposed to and the degree to which their fishing activities and associated livelihood are affected. Then, we investigated the adaptive actions that fishers have taken to cope with and adapt to the effects of climate change in addition to their adaptive capacity with respects to taking appropriate adaptive actions. Each indicator is given a weight/score by each individual fisher, and then all the individual scores of each indicator were computed and converted into an overall score or index for each component.

### Study area

**Figure 3:**
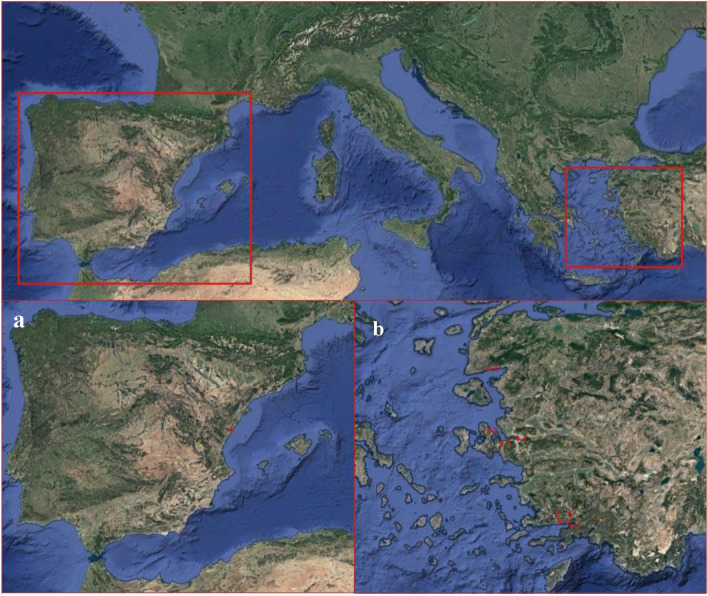
(**a**) Castelló de la plana’s location; (**b**) Fishery cooperatives data collected in the Aegean Sea: (1) Altınoluk, (2) Mordoğan, (3) Urla, (4) Bostanli, (5) Akbük, (6) Akyaka, (7) Akçapinar.

Two contrasting case studies were chosen to represent diverse fisheries and fishing practice in the Mediterranean Sea: one in the Western Mediterranean with different fleet characteristics and the other in the Eastern Mediterranean with small-scale fisheries dominated. The first case study is in Castelló de la Plana, Spain. This city is the capital of the province of Castelló and the region of La Plana Alta, which is in the Valencian Community (Fig. 3a). This region is characterized by a well-defined seasonality with relatively cold winters and hot summer periods, and this gradient marks the distribution of the fish species and their exploitation patterns. The most recent climate change is changing the “traditional” geographic distribution patterns of the fishes in the area^61^. In the western Mediterranean, the fishing fleets are heterogeneous and the fishing activity depends on multiple species, and many fishing fleets have adopted measures to ensure the sustainability of the fishery resources. For example, maximum daily/weekly catches (purse-seine fleet) or fishing 4 days per week (Trawling fleet).

Castelló is a rather small fishing community, but it is a representative of the similar problems that fishers have encountered in the Western Mediterranean region. In 2018 in the Castelló region, there were only 248 small-scaled fishers who were engaged in fishing activities, while a total of 64 fishing vessels engaged in fishing: 13 trawlers, 14 purse seiners, 2 pelagic longlines, and 34 small-scaled vessels. The fleet in the Castelló region mainly operates on the peninsula's continental platform in an area that has a width of 40 nautical miles (nmi) and a length of about 50 nmi. An exception (Trawling fleet) is applied to the fishing ban in the area at the bottoms of less than 50 m due to the large extension and low inclination of the peninsular platform, and fishing at more than 3 nmi is allowed even if the depth of 50 m is not reached. There is also a fishing closure season for 2 months a year, in summer for the trawling fleet and in winter for the purse-seine fleet, to protect the recruitment of some species.

The second case study is the Aegean Sea, where seven different fishery cooperatives were selected and analyzed along the coast of the Aegean Sea (Fig. 3b). The fisheries in this region are mainly dominated by small-scale fishing vessels. The fishing sector consists of a total of 4007 vessels, in which 96% of them (3836) are small-scale vessels. The number of the trawl vessels is only 54 and that of the purse seiners is 66^62^. The fishers in the area are usually organized under fishery cooperatives, and they use various gillnets, trammel nets, encircling nets, and longlines. There has been a decrease in the number of fishing vessels in the Aegean Sea due to the fishery buyback programs, overfishing, reduced catches, and the negative impacts of invasive species. It is unknown whether climate change had a direct impact on these changes or not. However, there was a remarkable decrease of 30% in the fishing fleet between 2008 and 2019. TUIK^62^ reported that the number of fishers who directly work on board is about 6542 in the Aegean Sea of Turkey, which is approximately 21% of the employed fishers on board of Turkish fishing fleets.

Several fishing cooperatives were selected based on their geographical locations which cover the whole coast of the Aegean Sea. They are representatives of small-scale fisheries with exhibited specific characteristics in the Eastern Mediterranean Sea (see Fig. 3). In addition, they were also chosen based on previous work and cooperation experience which make it relatively easier to get high and truthful responses. All the members of these cooperatives are artisanal fishers. Three cooperatives from the southern Aegean coast (Akyaka: 25 fishers, Akçapınar: 18 fishers, and Akbük: 11 fishers), three districts from the central Aegean coast (Mordoğan: 65 fishers, Urla: 33 fishers, and Bostanli: 157 fishers), and one from the northern Aegean region (Altınoluk: 117 fishers) were chosen as case studies.

### Indicator selection and data collection

#### Indicators

Each component of social vulnerability consists of a set of indicators. Most indicators were identified, selected and categorized based on literature^32,35–37,46,58,63–65^, and considered important components impacting fisheries vulnerability to climate change. However, some studies assess social vulnerability at a meso- or macro-scale (i.e. community, region and national) based on existing primary and secondary quantitative data^32,64^ while some studies assess social vulnerability on a micro-basis using household survey data and information^58,63,65^. This study employed the latter approach to assess social vulnerability at a micro level based on the data and information collected through a questionnaire survey with active fishers. The indicators for exposure are biophysical dimension, including temperature, storm, sea rise and ocean acidification while the indicators for sensitivity and adaptive capacity are derived from social, economic, fisheries, institutional and demographic dimensions. A total of 27 indicators (Table 1) were finally selected and categorized into different components based on the assessment framework.

#### Survey design and data collection

A semi-structured questionnaire was designed to include 35 multiple choice questions representing different dimensions and some basic information about the fishers. Each indicator corresponds to one of the questions in the questionnaire. Most questions have four answer choices, and one open answer if the answers were not covered. Some are composed of Yes or No choices. If the answer is Yes, then the likelihood (e.g., to what degree of changes happened) will be further asked by the interviewers (detailed description for each indicator is shown in Table 1). The surveys were carried out through face-to-face interviews with the fishers in the two study areas. Thus, it is easy to clarify, elaborate questions and record the answers. The questionnaire was tested with fishers when the first author worked as a fishery observer on an industrial trawler fishing demersal species outside the Canadian EEZ, Terranova Bank and Flemish Cup.

A total of 131 interviews were conducted: 46 interviews with fishers from Castelló area and 85 interviews with fishers from the Turkish part of the Aegean Sea. The 46 fishers in Castelló worked on different fishing vessels: 15 in trawling fleets, 15 in artisanal fleets, 15 in purse-seine fleets, and 1 in a pelagic long-line vessel. The interviews took place during the months of October and November 2018. In Turkey, the 85 interviews were carried out in three regions of the Aegean Sea (south, central, north) with their associated fishers’ cooperatives along the Aegean Sea coast between March and June of 2019. Among these surveys, 27 interviews in the Southern Aegean, 43 in the Central Aegean and 15 in the Northern Aegean region were conducted.

### Data analysis

#### Coding and indexing

Based on a four-point scale of low to very high defined^58,66,67^, the answer for each indicator from each fisher was indexed by a scale of 1–4, representing (1) Low, (2) Moderate, (3) High, and (4) Very High. The detailed classification of indicators was described in Table 1. Regarding the open question, it was not used due to no further information provided by fishers. For the climate stressors, only temperature and storm were used because other stressors were not chosen.

#### Data analysis

All the data from the survey were accordingly indexed, imported, and analyzed using Microsoft Office Excel and R psych-package. The first step is to apply Principal Component Analysis (PCA) to identify the most important indicators among variables from the survey with the standardized input indicators using the “psych” package (https://cran.r-project.org/web/packages/psych/psych.pdf) in the statistical software R-project. In the PCA framework, a loading value implies the correlation between an indicator and a component, representing the weight (coefficient) of each indicator for the component, i.e., how important each indicator to the component. The indicators that do not show correlation with any of the other indicators were screened out of the analysis^68,69^. For instance, the indicators such as the “Harvest species” and “Law” were removed from the analysis because they were not correlated with the rest of the indicators. The selected indicators to the Principal Component (PC1) explains at least 75% of the total variance^70^, and determines which indicator has the greatest relevance within each component. The weighted value for each indicator varied between − 1 and + 1. The sign (+ or −) of each indicator indicates the direction of its relationship with the other variables^71^. Each vector of loadings also defines the direction in space over which the variance of the data is greatest. Finally, the number of responses (46 *vs*. 85) differ between two regions, resulting in the different weight of the PC1 loadings. Therefore, the variance between two case studies differs, for instance, the variances for sensitivity and adaptive capacity in the Castelló are 0.68 and 0.82, respectively while they in the Aegean are 0.72 and 0.76, respectively.

The second analysis is to calculate the index of each indicator based on the four-point scale from low to very high. Each indicator was categorized as 4 for “very high (VH)”, 3 for “high (H)”, 2 for “moderate (M)” and 1 for “low (L)”. At the first step of the analysis, once the value of each indicator by each fisher is obtained, then employing Eq. (1)^58,67^calculates the average index of the indicators for all fishers and to obtain the value of each component within the vulnerability framework. The value of each component was then used to estimate the vulnerability (Table 3) for two different fishing regions using Eq. (2)^58^.

1 $$\text{I}=[\left(\text{L}\times 1\right)+\left(\text{M}\times 2\right)+(\text{H}\times 3)+(\text{V}\text{H}\times 4)]/(\text{L}+\text{M}+\text{H}+\text{VH})$$

2 $$\text{V}=(\text{E}+\text{S})-\text{AC}$$

where *I* is an index representing: *E* = Exposure; *S* = Sensitivity and *AC* = Adaptive Capacity. *L* = number of responses to the *low* indicator, *M* = number of responses to the *moderate* indicator, *H* = number of responses to the *high* indicator, and *VH* = number of responses to the *very high* indicator. We define the vulnerability levels based on index scores ranging from the lowest 0.00 to the highest 4.00 in this study. We further determine the score between 0.00 and 1.00 as low vulnerability, the score between 1.01 and 2.00 as moderate vulnerability, the score between 2.01 and 3.00 as high vulnerability and the score between 3.01 and 4.00 as very high vulnerability^66,72^.

- The study was approved by the Institutional review board of the Master Programme in Sustainable Fisheries Management at the University of Alicante.
- All methods were carried out in accordance with relevant guidelines and regulations.
- All participants provided informed consent to participate in this study.
